# Construction and Performance Study of a Dual‐Network Hydrogel Dressing Mimicking Skin Pore Drainage for Photothermal Exudate Removal and On‐Demand Dissolution

**DOI:** 10.1002/advs.202403362

**Published:** 2024-07-28

**Authors:** Xiaoxiao Ma, Lizhi Lin, Hang Luo, Qianqian Zheng, Hui Wang, Xiaoyan Li, Zhenfei Wang, Yongqiang Feng, Yu Chen

**Affiliations:** ^1^ School of Medical Technology Beijing Institute of Technology Beijing 100081 China; ^2^ Department of Polymer Science and Engineering Zhejiang University Zhejiang 310027 China; ^3^ School of Materials Science and Engineering Beijing Institute of Technology Beijing 100081 China; ^4^ Plastic Surgery Hospital of Peking Union Medical College and Chinese Academy of Medical Sciences Beijing 100144 China

**Keywords:** electrical stimulation, near‐infrared response, on‐demand dissolution, removal of exudate, wound dressings

## Abstract

In recent years, negative pressure wound dressings have garnered widespread attentions. However, it is challenging to drain the accumulated fluid under negative pressures for hydrogel dressings. To address this issue, this study prepared a chemical/physical duel‐network PEG‐CMCS/AG/MXene hydrogel composed by chemical disulfide crosslinked network of four‐arm polyethylene glycol/carboxymethyl chitosan (4‐Arm‐PEG‐SH/CMCS), and the physical network of hydrogen bond of agar (AG). Under near‐infrared light (NIR) irradiation, the PEG‐CMCS/AG/MXene hydrogel undergoes photothermal heating due to integrate of MXene, which destructs the hydrogen bond network and allows the removal of exudate through a mechanism mimicking the sweat gland‐like effect of skin pores. The photothermal heating effect also enables the antimicrobial activity to prevent wound infections. The excellent electrical conductivity of PEG‐CMCS/AG/MXene can promote cell proliferation under the external electrical stimulation (ES) in vitro. The animal experiments of full‐thickness skin defect model further demonstrate its ability to accelerate wound healing. The conversion between thioester and thiol achieved with L‐cysteine methyl ester hydrochloride (L‐CME) can provides the on‐demand dissolution of the dressing in situ. This study holds promises to provide a novel solution to the issue of fluid accumulations under hydrogel dressings and offers new approaches to alleviating or avoiding the significant secondary injuries caused by frequent dressing changes.

## Graphical abstract

This study prepares a chemical/physical duel‐network hydrogel composed by disulfide crosslinked network of 4‐Arm‐PEG‐SH/CMCS, and the network of hydrogen bond of agar. Under the photothermal effect, the hydrogel can not only effectively achieve the purpose of antibacterial, but also remove the excess exudate in the wound to prevent wound infection. Hydrogel dressings also have good electrical conductivity and on‐demand solubility.

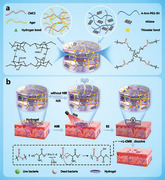

## 1 Introduction

Severe wounds, such as surgical trauma, burns, and diabetic ulcers, can generate significant amounts of wound exudate during the healing process. Failure to remove it can lead to high risks of wound infection.^[^ ^1^ ^]^ In clinic practice, negative pressure drainage is often used to remove the fluid accumulation under the dressing.^[^ ^2^ ^]^

Hydrogels have become the preferred material of moist dressings due to their advantages of strong hydrophilicity, good biocompatibility and similarity to the extracellular matrix.^[^ ^3^, ^4^ ^]^ However, the high water content of the conventional hydrogel dressing limits its exudate absorption capacity, which can lead to peri‐wound maceration and heightened risks of secondary infection, and subsequently affect the proliferation of wound‐rebuilding cells. In addition, the mechanical strengths of hydrogels are generally weak and become even weaker after fluid absorption. The negative pressure drainage often causes structural damages to the hydrogel dressing. These issues have become the critical bottlenecks that limit the widespread clinical application of hydrogel dressings.^[^ ^5^, ^6^ ^]^

Recently, researchers have shown extensive interests in the management and removal of wound exudate in the applications of hydrogel dressings. Various high absorption hydrogel dressings have been developed for excess exudate removal and wound healing promotion.^[^ ^7^ ^]^ Yet these solutions do not fundamentally remove the exudate and the excessive hydrogel swelling exerts pressures on the surrounding tissue, which not only affects adherence of the dressing on the wound, but also may increase the risk of infection.^[^ ^8^ ^]^ And self‐pumping oil‐water hydrogel dressings with excellent performances have attracted great attentions. This kind of dressings can rapidly remove the excess exudate with the efficiency ≈30 times greater than those of conventional pure hydrogel dressings, and can effectively promote the healing of burn wound.^[^ ^9^ ^]^

The skin can quickly open its pores to lower body temperature and create a suitable environment for survival through sweating upon subjecting to high temperatures.^[^ ^10^, ^11^ ^]^ Inspired by this sweating mechanism of the human skin, if we can construct a bioengineer responsive hydrogel with dynamic crosslinking reversible destructive properties under photothermal stimulation, the above problem of demand for the negative pressure drainage hydrogel dressing with excellent wound moisture management capability is promising to be solved. The natural polymer, agar, can exhibit excellent upper critical solution temperature (UCST) temperature‐sensitive effect. Its inherent physical hydrogen bond crosslinked structure can potentially enable the photo‐thermo‐reversible crosslinking effect. However, the stability of agar hydrogel constructed with a physical network is poor. Herein, a chemically crosslinked network was constructed via the sulfur bonds between 4‐Arm‐PEG‐SH and CMCS and then the physical hydrogen bond crosslinking network of agar was introduced to form a dual‐network structure. MXene was incorporated to enable the reversible crosslinking of agar by its photothermal effect, which could open the drainage pathway to mimic the one‐way drainage of skin pores. The introduction of MXene was also expected to provide the hydrogel with excellent electrical conductivity that could facilitate ES to accelerate wound healing. The chemically crosslinked structure with thioester‐thiol bonds could also offer the advantage of reversible dissolution of the hydrogel in the presence of L‐CME, potentially allowing for the on‐demand dressing removal from the wound surface to reduce secondary damage to the wound. The design of this dual‐network natural polymer hydrogel structure with the directional drainage function, healing promotion ability by ES, and on‐demand dressing dissolution is of significant value in enhancing the functional characteristics of hydrogel dressings to meet the wound repair demands.

## 2 Results and Discussion

### 2.1 Characterization of Hydrogel Structure

**Scheme 1:**
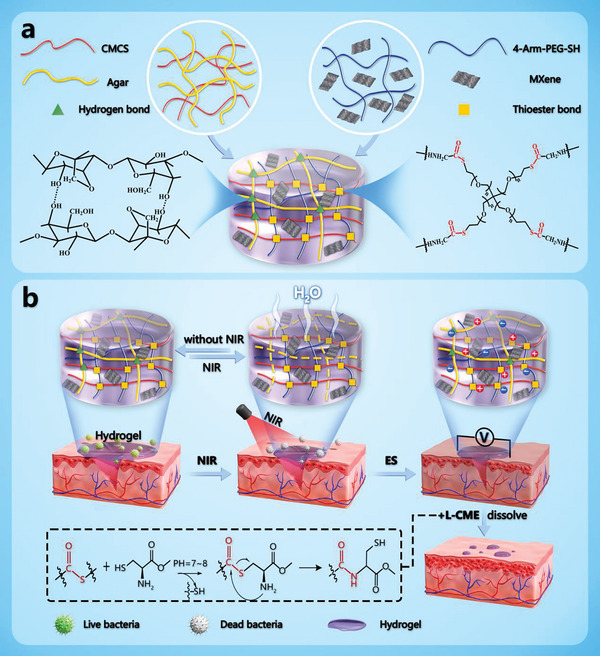
Hydrogel formation and wound repair mechanisms. a) In situ PEG‐CMCS/AG/MXene hydrogel formation mechanism; b) PEG‐CMCS/AG/MXene hydrogel wound repair efficacy design.

In this work, a chemically crosslinked network was constructed via the thiolation reaction between the thiol groups on 4‐Arm‐PEG‐SH and the carboxyl groups on CMCS at room temperature that formed a thioester structure.^[^ ^12^ ^]^ To achieve the directional drainage functionality in the hydrogel dressing, a hydrogen bond network of agar with the cooling crosslinking effect and UCST characteristics^[^ ^13^ ^]^ was constructed and combined with the PEG/CMCS network to form a PEG‐CMCS/AG dual‐network hydrogel with both chemically and physically crosslinked structures, as depicted in the formation mechanism (**Scheme** **1**a). The dual‐network structure was characterized with various analytical techniques including SEM, EDS, FTIR, and XPS to gain a comprehensive understanding.

#### 2.1.1 SEM

**Figure 1:**
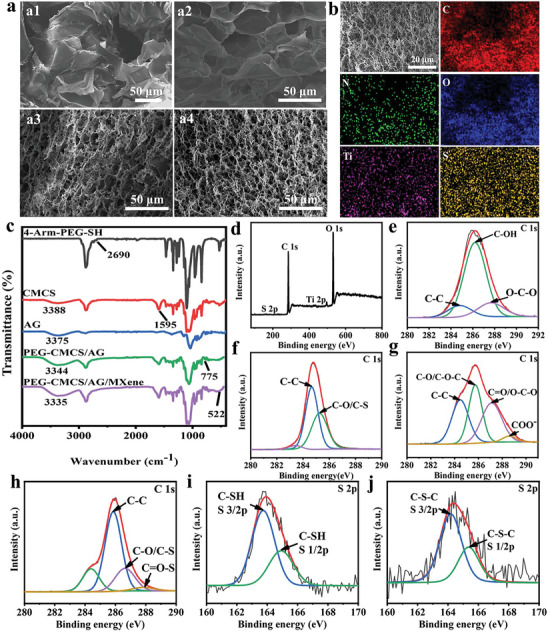
Structural characterization of hydrogel and its components. a) SEM images of AG hydrogel (a1), PEG‐CMCS hydrogel (a2), PEG‐CMCS/AG hydrogel (a3), and PEG‐CMCS/AG/MXene‐250 hydrogel (a4) sections; b) EDS mapping images of PEG‐CMCS/AG/MXene‐250 hydrogel; c) FTIR spectra of PEG‐CMCS/AG/MXene hydrogel and its components; d) XPS spectra of PEG‐CMCS/AG/MXene hydrogel; C1s spectra of AG e), 4‐Arm‐PEG‐SH f), CMCS g) and PEG‐CMCS/AG/MXene h); S2p spectra of 4‐Arm‐PEG‐SH i) and PEG‐CMCS/AG/MXene j).

The cross‐sectional morphologies of AG hydrogel, PEG‐CMCS hydrogel, PEG‐CMCS/AG hydrogel and PEG‐CMCS/AG/MXene‐250 hydrogel were imaged by SEM. As depicted in **Figure** **1**a, the pore diameters of AG and PEG‐CMCS hydrogel are about 150 µm and 30 µm, respectively. PEG‐CMCS/AG hydrogel is a well‐organized 3D porous structure with interconnected pores. Its network is mainly composed of two parts. One part is the main scaffold of the gel network formed via intra/intermolecular hydrogen bonds, which provides appropriate ion migration channels within the hydrogel. The other part consists of the structures with small pores formed by thioester bonds. The introduction of thioester bond reduces the pore diameters in the PEG‐CMCS/AG hydrogel due to the interaction between the physically and chemically crosslinked networks. The addition of MXene further decreases the pore diameters in the PEG‐CMCS/AG/MXene hydrogel because the hydrophilic functional groups of MXene tend to form hydrogen bonds with other components in the hydrogel network. The enhanced interaction between the dual‐network increases the crosslinking density, which provides a strong mechanical support for the hydrogel system. The abundant 3D porous structures offer pathways for the wound exudate absorption. Figure 1b shows the EDS spectrum of PEG‐CMCS/AG/MXene‐250 hydrogel. The distribution of Ti indicates that the MXene layers are evenly distributed on both the surface and within the hydrogel network structure.

#### 2.1.2 FTIR Spectra

The FTIR spectra of PEG‐CMCS/AG/MXene‐250 hydrogel and its components are shown in Figure 1c. The wide peak of AG at 3360 cm^−1^ is attributed to the stretching vibration of O─H.^[^ ^14^ ^]^ The absorption peak for ‐SH is observed at 2690 cm^−1^ in the spectrum of 4‐Arm‐PEG‐SH and the strong peak at 1100 cm^−1^ can be ascribed to the stretching vibration of C─O─C.^[^ ^12^ ^]^ The broad peak of CMCS at 3388 cm^−1^ is attributed to the stretching vibration of O‐H while the peak at 1595 cm^−1^ is fitted with ─COO^−^. The peak at 1342 cm^−1^ is associated with the symmetric stretching vibration of ─COO^−^.^[^ ^15^ ^]^ In the spectrum of PEG‐CMCS/AG, the enhanced broad peak at 3344 cm^−1^ is attributed to the stretching vibration of the hydrogen bonds formed by O‐H. The absorption peak of ‐SH disappears and a new peak appears at 775 cm^−1^ that can be assigned to C─S─C, indicating the formation of thioester bond.^[^ ^16^ ^]^ The characteristic peak of Ti‐O in MXene is observed at 522 cm^−1^ in PEG‐CMCS/AG/MXene‐250. Due to the presence of a large number of hydrophilic groups and carbonyl groups in MXene, the peaks caused by the stretching vibrations of carbonyl at 1591 cm^−1^ and hydroxyl group at 3335 cm^−1^ become significantly stronger in the composite hydrogel.

#### 2.1.3 X‐Ray Photoelectron Spectroscopy (XPS)

Figure 1d–j shows XPS spectra of AG, 4‐Arm‐PEG‐SH, CMCS, and PEG‐CMCS/AG/MXene hydrogels. The full scan XPS spectrum in Figure 1d reveals the presence of four elements, C, O, S and Ti, in the PEG‐CMCS/AG/MXene hydrogel. The C1s spectrum of AG consists of three distinct peaks at 284.82 eV, 286.21 eV and 287.66 eV, corresponding to the binding energies of C─C, C─OH and O─C─O, respectively (Figure 1e).^[^ ^15^ ^]^ The C1s peaks of 4‐Arm‐PEG‐SH at 284.66 eV and 285.25 eV are attributed to C─C and C─O/C─S, respectively (Figure 1f). In the C1s spectrum of CMCS depicted in Figure 1g, the carbon element exhibits four different chemical states at 284.92 eV, 286.25 eV, 287.58 eV and 288.68 eV, which correspond to the binding energies of C─C, C─OH, C═O and ─COO^−^ structures, respectively.^[^ ^17^ ^]^ The C1s peaks of PEG‐CMCS/AG/MXene hydrogel at 284.64 eV, 285.33 eV and 286.64 eV can be assigned to C─C, C─O/C─S and C═O─S, respectively (Figure 1h). As can be seen, the peak for C═O─S bond appears, as compared with the spectrum of 4‐Arm‐PEG‐SH, and the binding energies of both C─O and C═O bonds in CMCS decrease in the composite hydrogel, indicating the reaction between C═O bond and the sulfur. The C═O peak becomes weaker and the C─O peak becomes broader because of the hydrogen bonding between the polymers containing C─O. The S 2p spectrum of 4‐Arm‐PEG‐SH is deconvoluted into S 3/2p and S 1/2 of thiol group at 163.80 eV and 164.90 eV (Figure 1i), respectively.^[^ ^17^ ^]^ The binding energies of S 3/2p and S 1/2 increase to 164.13 eV and 165.33 eV in PEG‐CMCS/AG/MXene hydrogel due to the formation of C─S─C (Figure 1j).^[^ ^18^ ^]^ The presence of the thioester bond indicates the successful construction of thioester bond network.

### 2.2 Characterization of Physical and Chemical Properties

#### 2.2.1 Rheological Properties

**Figure 2:**
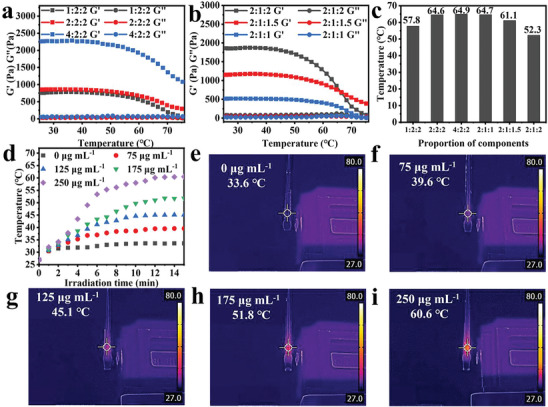
Characterization of physical and chemical properties of hydrogels. a,b) Rheological curves of the composite hydrogels with different 4‐Arm‐PEG‐SH:CMCS:AG ratios as the function of temperature; c) Transition temperatures of the composite hydrogels caused by hydrogen bond destruction within the temperature range of 25–80 °C; (d–i) Temperature changes of PEG‐CMCS/AG/MXene hydrogels during 15 min of irradiation with an 808 nm laser (1.5 W cm^−1^) d) and the infrared thermographic images (e–i) after the irradiation.

The introduction of MXene potentially enables the rapid temperature rising of the hydrogel under NIR irradiation, thereby achieving the dynamic drainage effect. Therefore, the thermosensitive properties of PEG‐CMCS/AG/MXene hydrogel were evaluated by the rheological test. The *G′* of the composite hydrogel shows a decreasing trend with the increasing of temperature, indicating the temperature dependent crosslinked structure destruction characteristics of the hydrogel. At the constant PEG‐CMCS content, increasing the AG content results in an upward trend in the *G′* of the composite hydrogel (**Figure** **2**a,b). It can be explained that more hydrogen bonds are formed at high AG content, resulting in stronger physical crosslinking effects and mechanical properties of the hydrogel. The intersection of the tangents to the rheological curve of *G′* before and after the inflection point represents the hydrogen bond destruction transition temperature (Figure 2c). With PEG‐CMCS content remained constant, the hydrogen bond destruction transition temperature gradually decreases with the increasing of AG content. The number of hydrogen bonds is more significantly affected by temperature at high AG contents, which weakens the interaction between the chemically crosslinked network and the physically crosslinked network. Therefore, the temperature required to destruct the crosslinking network is lower. The hydrogel hydrogen bond destruction transition temperature is the lowest at 4‐Arm‐PEG‐SH:CMCS:AG = 2:1:2. Therefore, the hydrogel prepared at this ratio is used for the subsequent experiments.

#### 2.2.2 Thermosensitive Properties

The thermosensitive properties induced by the physical hydrogen bonds within agar after NIR irradiation can assist in the dynamic drainage to maintain an appropriate environment for wound healing. Following the hydrogel preparation process in Section 4, 0.5 mL of PEG‐CMCS/AG/MXene composite hydrogels with different MXene contents were respectively injected into a glass tube using a twin‐screw syringe. The temperature in the hydrogel during the 15 min of irradiation with an 808 nm laser was monitored. As shown in Figure 2d, the temperature inside the composite hydrogel gradually rises as the MXene content increased due to the excellent photothermal conversion efficiency provided by its surface plasmon resonance (LSPR) effect. Specifically, the temperature inside the hydrogel gradually increases from 33.6 °C to 60.6 °C with the increasing of MXene content from 0 µg mL^−1^ to 250 µg mL^−1^ after the NIR irradiation. These results suggest that the higher the MXene content, the more pronounced the photothermal effect it exerts in the system. To maximize the inherent photothermal reversible drainage function of PEG‐CMCS/AG/MXene hydrogel, PEG‐CMCS/AG/MXene‐250 hydrogel prepared with 250 µg mL^−1^ MXene was used in subsequent experiments.

### 2.3 Photothermal Reversible Drainage Properties

**Figure 3:**
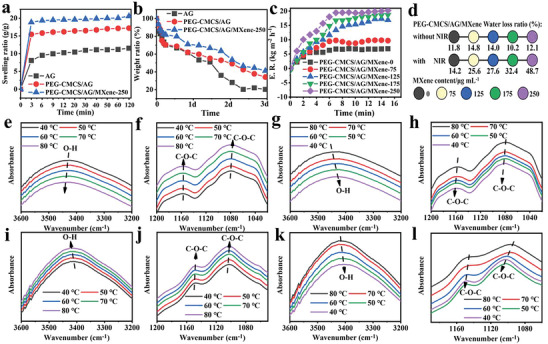
Photothermal reversible drainage performance of PEG‐CMCS/AG/MXene hydrogel and the relationship with the reversible crosslinking property. a) Swelling curves of AG hydrogel, PEG‐CMCS/AG hydrogel and PEG‐CMCS/AG/MXene‐250 hydrogel; b) Water retention rates of AG hydrogel, PEG‐CMCS/AG hydrogel and PEG‐CMCS/AG/MXene‐250 hydrogel after 3 days of storage at room temperature; c) Evaporation rates of PEG‐CMCS/AG/MXene hydrogels under he NIR irradiation at 1.5 W cm^−1^; d) Comparison of water loss rates of PEG‐CMCS/AG/MXene hydrogels with/without NIR irradiation; e–h) Infrared absorption peaks of hydroxyl and ether bonds in AG hydrogel as the functions of temperature, with (e,f) representing the changes during the heating process and (g,h) representing the changes during the cooling process; i–l) Infrared absorption peaks of hydroxyl and ether bonds in PEG‐CMCS/AG/MXene hydrogel as the functions of temperature, with (i,j) representing the changes during the heating process and (k,l) representing the changes during the cooling process.

Hydrogel dressings with high swelling capacities can adsorb more wound exudate to reduce the risk of wound infection and promote wound healing. The equilibrium swelling ratios of AG hydrogel, PEG‐CMCS/AG and PEG‐CMCS/AG/MXene hydrogel in 2 h are determined to be 12.2 g/g, 17.2 g/g, and 20.6 g/g, respectively, (**Figure** **3**a). Compared to single‐network hydrogel, the dual‐network hydrogels exhibit significantly higher swelling ratios. In particular, PEG‐CMCS/AG/MXene hydrogel reaches nearly 19 times its dry weight, approaching its equilibrium swelling ratio, in 3 min. It can be explained with the abundant hydrophilic functional groups and the hydrogen‐bond network structure in the hydrogel, which facilitates the infiltration of water molecules, and thus improves the swellability. The rapid and substantial wound exudate absorption can enable the initial step of the drainage process of the hydrogel itself.

Water‐retentive hydrogel dressings with good water‐holding capacities can prevent the dry necrosis of wound tissues, alleviate wound pain, promote the division and migration of epithelial cells, and expedite wound healing. The water retention rates of AG hydrogel, PEG‐CMCS/AG hydrogel and PEG‐CMCS/AG/MXene‐250 hydrogel at 30 °C in 3 d are determined as shown in Figure 3b. The water retention rate of PEG‐CMCS/AG/MXene‐250 hydrogel is the highest with the value of 41.1%, followed by PEG‐CMCS/AG hydrogel (34.1%), and that of AG hydrogel with the single network is the lowest with the value of 20.3%. The high water retention rate of PEG‐CMCS/AG/MXene‐250 hydrogel is due to its small pore sizes. In the PEG‐CMCS/AG/MXene hydrogel, the numerous hydrophilic functional groups in MXene itself can increase the number of hydrogen bonds and reduce the pore size in the hydrogel, as shown in SEM image in Figure 1a, which enhances the water retention performance. The excellent water retention properties of PEG‐CMCS/AG/MXene‐250 can provide the protection to the dressing during the wet wound healing process.

The evaporation rate (E. R.) of PEG‐CMCS/AG/MXene hydrogel increases from 7.0 kg m^−2^ h^−1^ to 20.2 kg m^−2^ h^−1^ with the increase of MXene content from 0 µg mL^−1^ to 250 µg mL^−1^ after the 15 min NIR irradiation (Figure 3c). The PEG‐CMCS/AG/MXene hydrogels show similar water loss rates without NIR irradiation, but the water loss rate increases from 14.2% to 48.7% with the increase of MXene content from 0 µg mL^−1^ to 250 µg mL^−1^ under the NIR irradiation (Figure 3d). The incorporation of MXene imparts excellent photothermal properties to the hydrogel, which heats the hydrogel, destructs the hydrogen bond network within the system, and facilitates the removal of excess exudate from the dressing under NIR radiation.

To further understand the influences of MXene incorporation and its photothermal effect on the hydrogen bond structure of PEG‐CMCS/AG/MXene hydrogel, temperature‐dependent FTIR spectra of agar hydrogel were measured. Figures 3e–h present the changes in the peaks of hydroxyl and ether bonds in the agar hydrogel during the temperature increase from 40 °C to 80 °C and decrease from 80 °C to 40 °C, respectively. As the temperature increased, the hydroxyl peak at 3429 cm^−1^ undergoes redshifts and the ether peaks at 1157 cm^−1^ and 1080 cm^−1^ blueshift, suggesting that high temperatures can destruct the hydrogen bond structure.^[^ ^19^ ^]^ Accordingly, as the temperature decreased from 80 °C to 40 °C, the hydroxyl peak at 3448 cm^−1^ blueshifts and the ether peaks at 1156 cm^−1^ and 1079 cm^−1^ redshift, indicating the recovery of the hydrogen bond network.^[^ ^20^ ^]^ Figure 3i,l show the temperature dependent infrared spectra of PEG‐CMCS/AG/MXene hydrogel. Similarly, the hydroxyl peak redshifts from 3411 cm^−1^ to 3418 cm^−1^ and the aliphatic ether peak at 1149 cm^−1^ and the aromatic ether peak at 1104 cm^−1^ both blueshift as the temperature increased from 40 °C to 80 °C, indicating the destruction of the hydrogen bond network. As the temperature decreased from 80 °C to 40 °C, the hydroxyl peak at 3419 cm^−1^ blueshifts and the aliphatic ether peak at 1144 cm^−1^ and the aromatic ether peak at 1040 cm^−1^ experiences redshifts, indicating the recovery of the hydrogen bond network. These results indicate that the hydrogen bond network structure formed by the hydroxyl and ether groups of agar is destructed in the PEG‐CMCS/AG/MXene hydrogel due to the photothermal effect. Consequently, the degree of crosslinking is reduced, which can open the channels for water molecules to evaporate for the excess water and exudate removal from the dressing. The restoration of the hydrogen bond network increases the degree of crosslinking of the PEG‐CMCS/AG/MXene hydrogel as the temperature decreased, which closes off the pathways for water molecule evaporation and allows for the maintenance of a moist wound environment. This photothermal effect of the hydrogel can maintain an appropriately moist environment around the wound site during the practical applications and prevent the negative effects on wound healing that arises from excessive humidity or gel desiccation. The chemically crosslinked network structure of thioester bond in the dual network can maintain the structural and mechanical integrity of the hydrogel, even after the destruction of the hydrogen bond structure, which ensures the stability for its practical application on wounds.

### 2.4 In Vitro Biological Performances

#### 2.4.1 In Vitro Antibacterial Activity

**Figure 4:**
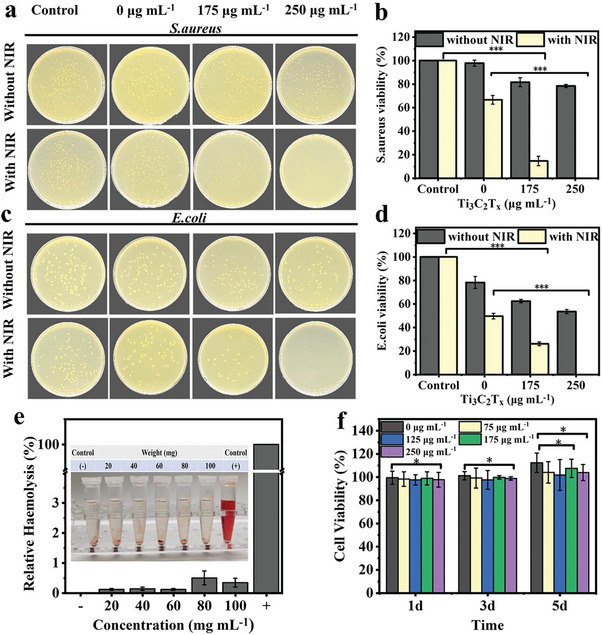
In vitro biological characterization of hydrogels. a,b) Images and quantitative analysis of *S. aureus* on PEG‐CMCS/AG/MXene hydrogels with/without NIR irradiation; c,d) Images and quantitative analysis data of *E. coli* on PEG‐CMCS/AG/MXene hydrogels with/without NIR irradiation; e) Hemolysis rates and images of hemolysis testing of PEG‐CMCS/AG/MXene hydrogels; f) Cell viabilities of L929 cells cultured with PEG‐CMCS/AG/MXene hydrogels leachate for 1 d, 3 d, and 5 d for cytotoxicity evaluation (n = 3, mean ± SD, *p < 0.05, **p < 0.01, and ***p < 0.001).

During the healing process, the wound is susceptible to bacterial infections, which can even lead to infection‐related complications.^[^ ^21^ ^]^ Therefore, the current focus in wound management is on the utilization of wound dressings to prevent and control infections and create a microenvironment conducive to wound healing. **Figure** **4**a–d display the antimicrobial activity assay results of PEG‐CMCS/AG/MXene hydrogel against *Staphylococcus aureus* (*S. aureus*) and *Escherichia coli* (*E. coli*). In the absence of NIR irradiation, the bactericidal rate against *S. aureus* rises from 0% to 21.6% and that against *E. coli* increases from 0% to 46.5% after MXene introduced, suggesting MXene can bring certain bactericidal activities to the hydrogel.^[^ ^22^ ^]^ Under NIR irradiation, the bactericidal rates of hydrogel against *S. aureus* and *E. coli* reach 100% as the MXene content increased. Therefore, MXene improves the antibacterial performance of the hydrogel via two mechanisms. First, its hydrophilic functional groups make its adsorption on the surfaces of bacteria very easy. The adsorption deactivates the microorganisms and blocks the nutrient supply of the bacteria. Second, the photothermal heating effect causes damages to the cells and eventual the cell death under NIR irradiation. Therefore, the antibacterial activity of PEG‐CMCS/AG/MXene hydrogel is attributed to the synergistic effect of the structure of MXene itself and its photothermal property. Such excellent antibacterial activity can reduce the inflammation caused by bacterial infection and promote wound healing.

#### 2.4.2 In Vitro Biosafety

Good biocompatibility is essential for the clinical application of a wound dressing to avoid inhibiting the growth and function of healthy cells and destructing the physiological healing processes. Considering the application purpose involves the direct contact with blood, blood compatibility testing was first conducted. As shown in Figure 4e, unlike the positive control group, the red blood cell solutions containing different amounts of PEG‐CMCS/AG/MXene‐250 hydrogel remain almost transparent. The hemolysis rates with different amounts of PEG‐CMCS/AG/MXene‐250 hydrogel are all below 1%, lower than the international medical material standard of 5%,^[^ ^23^ ^]^ demonstrating the excellent blood compatibility of the hydrogel.

Figure 4f shows the cytotoxicity test results of PEG‐CMCS/AG/MXene hydrogel. L929 cells were incubated with the PEG‐CMCS/AG/MXene hydrogel leachate for 1 d, 3 d, and 5 d, respectively, and the cell viabilities were determined by the CCK‐8 method. The results indicate that the cell viabilities of all tests with different PEG‐CMCS/AG/MXene hydrogels remain above 80%, and even increase after 3 d and 5 d of culture, validating the good biocompatibility of the hydrogel.

#### 2.4.3 Conductivity and Effect on Cell Proliferation under ES In Vitro

**Figure 5:**
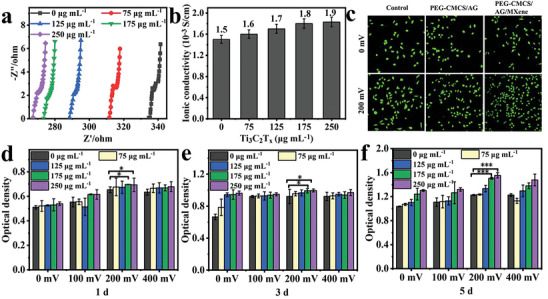
Electrical conductivity of the hydrogel and its influence on cell proliferation in vitro. a) Impedance spectra of PEG‐CMCS/AG/MXene hydrogels; b) Ionic conductivities of PEG‐CMCS/AG/MXene hydrogels; c) Live cell staining of L929 cells cultured after receiving ES of 0 mV and 200 mV for 3 d; d–f) Cell viabilities of L929 cells cultured on PEG‐CMCS/AG/MXene hydrogels and exposed to different voltages (0‐400 mV) for different durations (1 d, 3 d, and 5 d) (n = 5, mean ± SD, *p < 0.05, **p < 0.01, ***p < 0.001).

As composited into the PEG‐CMCS/AG hydrogel, MXene also shows noteworthy influences on the hydrogel electrical conductivity. **Figure** **5**a shows the AC impedance spectra of PEG‐CMCS/AG/MXene hydrogels at the frequency of 10^3^–10^5^ Hz. As can be seen, the impedance gradually decreases and the conductivity gradually increases with the increase of MXene content (Figure 5b). The high conductivity of PEG‐CMCS/AG/MXene is attributed to the free movements of sodium ions in the electric field. The composition of the layered structure of MXene nanomaterial increases the pathways for ion mobility, which facilitates the ion movement, reduces ion transport resistance, and thus enhances the ionic conductivity in the hydrogel.

Cells communicate with each other through bioelectrical signals. It is known that ES can promote cell division and proliferation.^[^ ^24^ ^]^ Figure 5c shows the live cell staining images of L929 cells on the PEG‐CMCS/AG hydrogel and PEG‐CMCS/AG/MXene‐250 hydrogel before and after receiving 0 mV and 200 mV ES for 3 d. After 200 mV electrical stimulation, the cell activity of PEG‐CMCS/AG/MXene group was higher than that of PEG‐CMCS/AG group and control group, indicating that electroactive PEG‐CMCS/AG/MXene hydrogel could enhance the proliferation activity of L929 cells under ES. Next, quantitative study was conducted on the proliferative activities of L929 cells on the PEG‐CMCS/AG/MXene hydrogels under ES in the range from 0 mV to 400 mV (Figure 5d–f). The OD value of L929 cells increases faster at higher MXene contents under the same ES, suggesting that the enhancement of hydrogel conductivity promotes cell activity. The highest OD value is observed at the ES voltage of 200 mV, suggesting that the appropriate level of ES can enhance cell proliferative activity and excessive ES can inhibit cell proliferation. In all, the PEG‐CMCS/AG/MXene hydrogel can enhance the proliferative activity of L929 cells upon proper ES.

### 2.5 On‐Demand Hydrogel Dissolution

**Figure 6:**
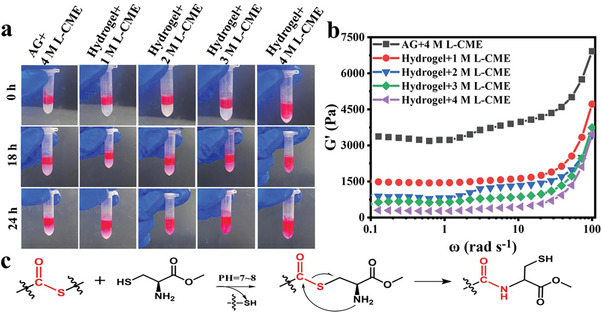
Dissolutions of AG hydrogel and PEG‐CMCS/AG hydrogel in different concentrations of L‐CME solutions. a) Photos of the dissolutions of hydrogels in different concentrations of L‐CME aqueous solutions over 24 h; b) Rheological diagrams of AG hydrogel and PEG‐CMCS/AG hydrogel soaked in L‐CME solutions for 24 h; c) The thioester‐thiol conversion mechanism for the on‐demand dissolution of PEG‐CMCS/AG hydrogel.

The strong adhesive properties of a dressing can sometimes cause to the secondary damages to the wound during the dressing removal. PEG‐CMCS/AG hydrogel contains thioester bonds that can potentially be destructed by the S_N_2 nucleophilic substitution reactions with the compounds containing ‐SH groups to enable the on‐demand dressing dissolution in situ, and thus avoid secondary harm to the patient during the removal process.^[^ ^25^ ^]^ Herein, the solution of a typical compound, L‐CME, with excellent biocompatibility and ‐SH functional groups, was chosen to explore the dissolubility performance of PEG‐CMCS/AG hydrogel. As shown in **Figure** **6**a, the hydrogel shows no obvious dissolution trend within 24 h and maintains its original appearance in 1 M L‐CME solution, is partially dissolves as the L‐CME concentration increased to 2 M and 3 M, but is completely dissolved after soaked in 4 M L‐CME solution for 24 h. The AG hydrogel is completely insoluble even after immersed in 4 M L‐CME for 24 h. To further understand the structural changes of the hydrogel during the dissolution, the hydrogel discs with the diameters of 2 cm and thicknesses of 2.5 mm were immersed in the L‐CME solutions of different concentrations (1 M, 2 M, 3 M and 4 M) at room temperature for 24 h and their rheological curves were measured. As can be seen from Figure 6b, the energy storage modulus of PEG‐CMCS/AG/MXene hydrogel decreases from 1507 Pa to 290 Pa with the increase of L‐CME solution concentration from 1 M to 4 M. In contrast, the storage modulus of AG hydrogel remains a significantly higher level than that of PEG‐CMCS/AG/MXene hydrogel after immersed in 4 M L‐CME for 24 h. These results further confirm that the L‐CME solution can destruct the thioester bond network formed with 4‐Arm‐PEG‐SH, but show no significant effects on the hydrogen bond network.

Figure 6c shows the structural transformation mechanism of thioester‐thiol bonds in L‐CME solution. Due to the strong nucleophilic properties of its ‐SH, L‐CME can promote the conversion of thioester‐thiol bonds via the S_N_2 nucleophilic substitution reaction to destruct the crosslinked structure of the hydrogel.^[^ ^26^, ^27^ ^]^ Simultaneously, the lone pair of electrons in the ‐NH_2_ of L‐CME undergo intramolecular electron transfer via nucleophilic action, which exposes the thiol bonds at the end of the molecular chain structure and breaks the original thioester‐thiol bond equilibrium.^[^ ^28^ ^]^ Therefore, 4 M of L‐CME solution can be applied on the hydrogel dressing surface during the self‐dissolving debridement stage. The crosslinked structure is then removed through the thioester‐thiol conversion, realizing the in situ dissolution of dressing and reducing the secondary damage caused by the dressing removal.^[^ ^29^ ^]^

In the PEG‐CMCS/AG/MXene hydrogel, the first network consisting of thioester bonds imparts the hydrogel with ionic conductivity and on‐demand dissolution capability. The second network composed of the hydrogen bonds between AG molecules ensures the good mechanical and thermosensitive properties of the hydrogel.^[^ ^30^ ^]^ The incorporation of MXene with high thermal conductivity into the PEG‐CMCS/AG hydrogel aims to take the advantage of its photothermal effect under NIR irradiation which can raise the temperature of the system and destruct the hydrogen bond structure. The destruction results a “channel opening” effect, which facilitates the removal of excess exudate during wound healing and allows for a pain‐free removal process. MXene can also increase the conductivity of the hydrogel, which promotes cell proliferation and is promising to accelerate wound healing under ES. In addition, the in situ dissolution of PEG‐CMCS/AG/MXene hydrogel dressing can be achieved by the conversion of the thioester bond to thiol bond using L‐CME, which holds the promise of avoiding secondary harm to patients during the dressing removal. Scheme 1b shows the efficacy design of PEG‐CMCS/AG/MXene hydrogel for wound repair.

### 2.6 Application of PEG‐CMCS/AG/MXene Hydrogel to Wound Treatment In Vivo

**Figure 7:**
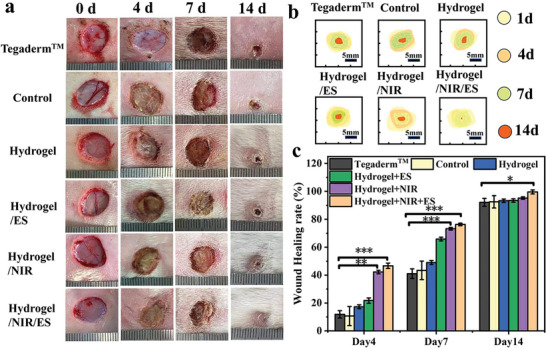
Wound treatment effects of different dressings. a) Photos of the wounds treated with different dressings on different treatment days; b) Wound closure traces of different treatments; c) Comparison of wound areas of different groups at different time points. (n = 3, mean ± SD, *p < 0.05, **p < 0.01, and ***p < 0.001).

Different dressings were applied to the wounds on the New Zealand rabbits with the full‐thickness skin defect model. The wound treatments are divided into six groups: Tegaderm^TM^, Control, PEG‐CMCS/AG/MXene, PEG‐CMCS/AG/ES, PEG‐CMCS/AG/NIR and PEG‐CMCS/AG/NIR/ES groups. The PEG‐CMCS/AG/MXene/NIR group was irradiated with an 808 nm laser for 10 min on daily basis and the PEG‐CMCS/AG/MXene/ES group was stimulated with 200 mV voltage on the hydrogel for 10 min every day. The PEG‐CMCS/AG/MXene/NIR/ES group was subjected to both NIR irradiation and ES, each for 10 min every day. The wounds were imaged on day 0, 2, 7, and 14 and analyzed to assess the wound healing progresses. **Figure** **7**a–c clearly show that the wound closure rate of the PEG‐CMCS/AG/MXene group is significantly higher than those of the Tegaderm^TM^ group and the Control group by day 14, which can be explained with the excellent wound affinity of the hydrogel. The wound healing rates of the PEG‐CMCS/AG/MXene/ES and PEG‐CMCS/AG/MXene/NIR groups reach 93.4% and 95.2%, respectively, by day 14, indicating either ES or NIR can promote wound healing. The electrical activity of the hydrogel under ES can enhance the intercellular communication, thereby increasing the wound healing efficiency. The photothermal reversible dynamic dehydration effect of the hydrogel can promote the evaporation of wound exudate under NIR irradiation to maintain an appropriate healing environment. The photothermal heating effect also endows the hydrogel good bactericidal activity, which further benefits the wound healing process. The PEG‐CMCS/AG/MXene/NIR/ES group shows an impressive wound healing rate of 99.6% with the wound almost completely healed, demonstrating the advantages of the synergistic effect of NIR and ES in promoting tissue regeneration. The strong antibacterial activity and good conductivity of PEG‐CMCS/AG/MXene/NIR/ES hydrogel can promote the wound healing and its ability to promptly remove wound exudate can maintain a proper moist environment for wound healing. Therefore, the CMCS/AG/MXene/NIR/ES treatment can significantly accelerate wound healing.

**Figure 8:**
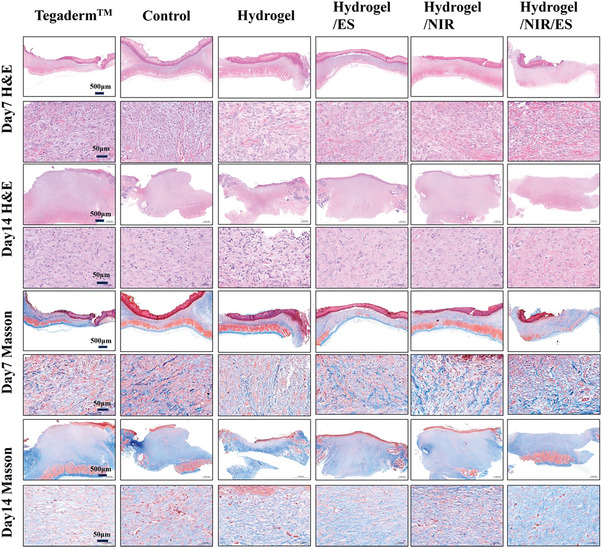
H&E staining and Masson staining of the tissues of different treatment groups collected on day 7 and 14.

To further assess the efficiencies of different treatment conditions, the wound tissues collected on day 7 and 14 were subjected to H&E staining and Masson staining for histological analysis. As shown in **Figure** **8**, all groups exhibit inflammations in the wound by day 7, yet the Control group and Tegaderm^TM^ group show the deepest purple color, indicating the most significant inflammation. The severe wound infections make the recoveries of these two groups the slowest.^[^ ^14^ ^]^ The wounds of PEG‐CMCS/AG/MXene/NIR and PEG‐CMCS/AG/MXene/ES groups are also purple, but lighter. Some fibroblasts are generated and the wound recovery is still slow. The inflammation of the PEG‐CMCS/AG/MXene/NIR/ES group is the mildest with re‐epithelialization, and the regenerated tissue structure is clearly observed. By day 14, the epithelial regenerations of Control group and the Tegaderm^TM^ group are incomplete, and small numbers of fibroblasts with disordered arrangements are generated, yet with a more significant inflammatory response.^[^ ^31^ ^]^ The PEG‐CMCS/AG/MXene group exhibits more pronounced inflammations. NIR or ES promotes the healing progress in the PEG‐CMCS/AG/MXene/NIR and PEG‐CMCS/AG/MXene/ES groups with the formation of both epidermal and dermal layers on the wound. The wound healing of the PEG‐CMCS/AG/MXene/NIR/ES group is the most complete due to the combined action of NIR and ES. The epidermis and connective tissue are more regular, and the fibroblast density is high. Compared with those of other groups, the wound fibers in the PEG‐CMCS/AG/MXene/NIR/ES group are a more organized tissue structure.^[^ ^32^ ^]^ These results further demonstrate that the synergistic effect of ES and NIR brings the best wound healing efficiency to the hydrogel.

**Figure 9:**
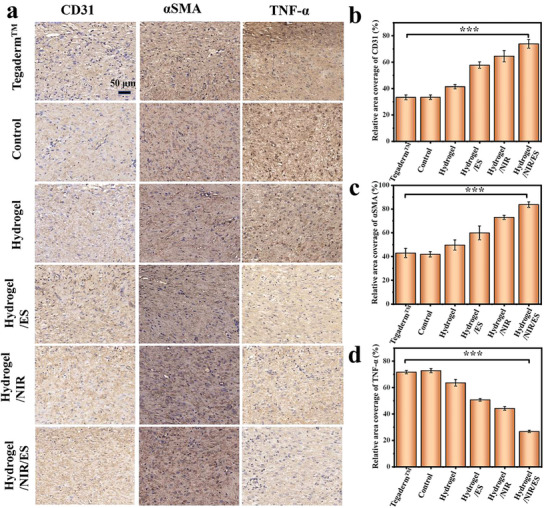
Immunohistochemical staining images and quantitative analysis results of different treatment groups on day 14. a) Immunohistochemical staining images against CD31, αSMA and TNF‐α; b–d) Quantitative analysis of the CD31(b) αSMA (c) and TNF‐α (d) expression levels in different groups.

The wound healing efficiencies of different groups were further assessed by immunohistochemical staining to gain a deeper understanding of the regeneration of epidermis on wound. **Figure** **9**a displays the immunohistochemical staining images of the skin wound tissues of different groups collected on day 14 of the treatments against CD31, αSMA, and TNF‐α. Figure 9b–d show the corresponding quantitative analysis results of their expression levels. The staining against CD31 assesses the vascularization in the wound healing area during the regeneration process using vascular density.^[^ ^33^ ^]^ Figure 9a,b clearly show a significantly larger number of blood vessels in the PEG‐CMCS/AG/MXene/NIR/ES group, as compared with those of the Control and Tegaderm^TM^ groups. Myofibroblast (αSMA) is considered as a marker protein of myofibroblasts and it plays an important role in tissue fibrosis. As shown in Figure 9a,c, the expression levels of αSMA in Control, Tegaderm^TM^ and PEG‐CMCS/AG/MXene groups are low and that in PEG‐CMCS/AG/MXene/NIR/ES group is the highest. Locally released cytokines can activate the innate immune system to regulate the wound healing process during the inflammation stage. Among these cytokines, TNF‐α is one of the earliest and most significant pro‐inflammatory cytokines to appear in the inflammatory response.^[^ ^34^, ^35^ ^]^ It is evidenced from Figure 9a that the expression levels of TNF‐α in Control and Tegaderm^TM^ groups are the highest, indicating the highest levels of inflammation of these groups. The expression level of PEG‐CMCS/AG/MXene/NIR/ES group is the lowest, suggesting its least inflammation (Figure 9d). These results suggest that the PEG‐CMCS/AG/MXene/NIR/ES treatment can promote angiogenesis, reduce the expression of the pro‐inflammatory factor TNF‐α, and simultaneously upregulate the productions of CD31 and αSMA. Therefore, it can greatly promote wound healing and is more effective than other treatments.

## 3 Conclusion

This study aimed to address the critical issues of secondary infection caused by exudate accumulation during the wound treatment using hydrogel dressings and the challenges in managing and transferring wound exudate through negative pressure drainage. A dual‐network natural polymer hydrogel, PEG‐CMCS/AG/MXene, with the drainage effect mimic the function of skin pores was constructed based on the thioester bonds formed between 4‐Arm‐PEG‐SH and CMCS, the UCST thermosensitive effect of agar and the excellent photothermal effect of MXene. The physically/chemically crosslinked dual‐network hydrogel can be formed by the in situ gelation method. The characterizations by rheology, thermal imaging and variable‐temperature infrared spectroscopy demonstrate that the photothermal effect of PEG‐CMCS/AG/MXene hydrogel can destruct the hydrogen bond network under NIR irradiation, showing a photothermal dehydration effect to facilitate wound exudate removal. The hydrogen bond network is restored as the temperature returns to normal, which maintains a moist environment for the wound healing. The photothermal effect simultaneously enhances the antibacterial activity of the hydrogel to 100% against *S. aureus* and *E. coli*. The introduction of MXene increases the ionic conductivity of hydrogel up to 1.9 × 10^−3^ S cm^−1^, which enables the ability of the hydrogel to actively regulate the cell behavior under ES stimulation to promote wound healing. The in vivo experiments using a full‐thickness skin defect model on New Zealand rabbit further demonstrate that the PEG‐CMCS/AG/MXene hydrogel can achieve a high wound healing rate of 99.6% under the dual actions of NIR and ES. The PEG‐CMCS/AG/MXene hydrogel dressing can slowly dissolve in 4 M L‐CME, which allows for the self‐dissolution of the dressing to avoid secondary harm to the wound during the dressing removal. In all, our work provides a novel approach to the development of dual‐network hydrogel dressings with photothermal directional drainage capabilities, healing promotion responses to electrical stimulation, on‐demand dissolution by mimicing the exudate management effect of skin pores. It holds great potentials for the clinical application as a wound dressing material.

## 4 Experimental Section

### Materials

The four‐arm polyethylene glycol (4‐Arm‐PEG‐SH, analysis grade) was purchased from Xiamen Saenobio Biotech Co., Ltd. Low melting point agar (AG, analytical grade) was purchased from Shanghai Aladdin Biochemical Technology Co., Ltd. Carboxymethyl chitosan (CMCS, viscosity ≤ 80 mPa s; degree of carboxymethylation, 84%) was obtained from Zhejiang Aoxing Biotechnology Co., Ltd. L‐Cysteine methyl ester hydrochloride (L‐CME, purity 98%) was purchased from Shanghai Maclin Biochemical Co., Ltd. Ti_3_C_2_T_x_ was prepared using an acid etching method and the detailed procedure could be found in Figure S1 (Supporting Information). LiF and Ti_3_AlC_2_ were purchased from Shanghai Macklin Biochemical Co., Ltd. HCl and anhydrous ethanol were both purchased from Bei Jing TongGuang Fine Chemicals Company. The dye Rose Bengal sodium salt (85%) was purchased from Ruibio, Germany. Agar powder, peptone, sodium chloride, yeast extract, high glucose DMEM medium, phosphate‐buffered saline (PBS), fetal bovine serum (FBS), and the cell counting kit‐8 (CCK‐8) were all purchased from Beijing Solaibao Technology Co., Ltd. *Staphylococcus aureus* (*S. aureus*) and *Escherichia coli* (*E. coli*) were obtained from the National Center for Nanoscience and Technology (Beijing, China).

### Preparation of Hydrogels

First, 2 wt% of agar powder was dissolved in 1 mL deionized water and sufficiently stirred at 90 °C for 2 h until the solution became clear and transparent. Subsequently, 1 wt% of CMCS powder was added into the agar solution and agitated for 30 min until it was completely dissolved. 4‐Arm‐PEG‐SH (2 wt%) was dissolved in 1 mL MXene suspension (0, 75, 125, 175, 250 µg mL^−1^, respectively) and shaken at room temperature. Finally, the two solutions were evenly mixed and poured into a dish, and cooled to obtain PEG‐CMCS/AG/MXene hydrogel. PEG‐CMCS hydrogel, AG hydrogel, and PEG‐CMCS/AG hydrogel were prepared using the same process with the corresponding ingredient compositions.

### Determination of on‐Demand Dissolution of PEG‐CMCS/AG/MXene Hydrogel

250 µL of L‐CME solutions of different concentrations (1 M, 2 M, 3 M, 4 M) in PBS buffer with the pH of 7.2 – 7.4 were prepared, and respectively mixed with 50 µL 0.05 mg mL^−1^ Rose Bengal sodium salt. The mixtures containing the dye were respectively combined with the preformed PEG‐CMCS/AG/MXene and AG hydrogels, and the gel dissolution process was monitored for 24 h.

### Scanning Electron Microscopy (SEM)

After sputter‐coating the freeze‐dried hydrogel sample section with gold, the sectional morphology was imaged using a scanning electron microscope (S‐4800, Hitachi, Japan) at the voltage of 5 kV. The surface elemental composition was determined by energy‐dispersive X‐ray spectroscopy (EDS) at the testing voltage of 10 kV and the current of 30 µA.

### Fourier Transform Infrared Spectroscopy (FTIR) and Variable Temperature Infrared Spectroscopy

The chemical structures of the raw materials including 4‐Arm‐PEG‐SH, CMCS, and agar powder were first characterized by FTIR using a Bruker 80 V infrared spectrometer (Bruker, Germany) in the scanning range of 400–4000 cm^−1^ at the resolution of 4 cm^−1^. Variable temperature infrared spectroscopy was conducted to monitor the changes in the hydrogen bonds with temperature in AG hydrogel and PEG‐CMCS/AG/MXene hydrogel. The temperature was varied in the range of 40–80 °C at the heating rate of 1 °C min^−1^. Data were collected after each preset temperature was reached and maintained for 10 min.

### X‐Ray Photoelectron Spectroscopy (XPS)

XPS analysis of 4‐Arm‐PEG‐SH, CMCS, AG, and PEG‐CMCS/AG/MXene was performed on PHI QUANTERA‐II SXM X‐ray photoelectron spectrometer (Ulvac‐Phi, Japan) with an Al Ka X‐ray source. Broad‐spectrum scans were conducted in the range of 0 to 1100 eV.

### Rheological Properties

The temperature‐dependent rheological properties of the hydrogel were characterized using an MCR320 rheometer (Anton Paar, Austria). The changes in storage modulus (*G′*) and loss modulus (*G*) of the hydrogel were recorded at the frequency of 1 Hz and the constant normal force (F_N_) of 1 N in the temperature range of 25–80 °C to understand its thermosensitive properties.

### Swelling Properties

The discs of dried AG hydrogel, PEG‐CMCS/AG hydrogel and PEG‐CMCS/AG/MXene hydrogel discs (diameter 13 mm, thickness 2 mm) were immersed in a PBS solution (10 mM, pH = 7.4) to determine their swelling ratios,^[^ ^36^ ^]^ as described in Figure S2.3.1 (Supporting Information)

Water retention properties: Circular hydrogel discs with the diameters of 8 mm and thicknesses of 4 mm were prepared to assess the moisture retention performances of AG hydrogel, PEG‐CMCS/AG hydrogel and PEG‐CMCS/AG/MXene hydrogel. The discs were placed in an environment of 30 °C and 40% relative humidity for specified durations. The weights of the discs were recorded at the preset intervals. Three parallel tests were conducted for each sample. The water retention rate (*WR_i_*) was calculated using the formula as follows.

(1) $$WR_{i}\left(\%\right)=\frac{W_{i}}{W_{0}}\times 100\%$$

where *W_i_* was the weight of the hydrogel disc at different time points, and *W_0_* was the initial weight of the hydrogel disc.

Photothermal reversible drainage properties: To determine the photothermal performances of PEG‐CMCS/AG/MXene hydrogel, it was irradiated with a near‐infrared laser (808 nm, 1.5 W cm^−2^, Beijing TopView Technology Co., Ltd., China) for 15 min. The temperature changes were recorded using an infrared thermal imaging camera (FLIR E5, FLIR Systems, USA), and the equilibrium mass of the hydrogel was continuously monitored. The evaporation rate (E.R.) of the composite hydrogel was calculated as detailed in Figure S2.3.2 (Supporting Information).

### Conductivity Measurement

The electrical conductivities of PEG‐CMCS/AG/MXene hydrogels with different MXene contents were measured by the alternating current impedance method using an electrochemical workstation CHI760E (Shanghai Chenhua Instrument Co., Ltd., China). The detailed test procedure and calculation method were detailed in Figure S2.3.3 (Supporting Information).^[^ ^37^ ^]^

### Antibacterial Activity

The antibacterial activities of the hydrogels with different MXene contents against the Gram‐negative bacterium *E. coli* and Gram‐positive bacterium *S. aureus* were assessed by the colony counting method.^[^ ^21^ ^]^ The detailed procedure can be found in Figure S2.4.1 (Supporting Information). All samples were divided into the groups with/without NIR irradiation. The NIR irradiation groups were irradiated under the near‐infrared 808 nm laser (1.5 W cm^−2^) for 10 min after contacted with the bacterial suspension.

### Hemolysis Rate Test

Hemolysis rate was one of the parameters used to assess the safety of a medical material and its suitability in clinical applications.^[^ ^38^ ^]^ The hemolytic performance of the hydrogel was determined by measuring the absorbance of the mixed solution of red blood cells and the hydrogel at 540 nm using a microplate reader. The hemolysis rate was calculated following the detailed procedure in Figure S2.4.2 (Supporting Information).

### Cytotoxicity Test

The cell viability of mouse fibroblasts (L929) in response to the leachate of 0.1 g mL^−1^ hydrogel was determined by the CCK‐8 (Cell Counting Kit‐8) assay to assess the cytotoxicity of PEG‐CMCS/AG/MXene hydrogel.^[^ ^39^ ^]^ The detailed procedure can be found in Figure S2.4.3 (Supporting Information).

### In Vitro Experiments for Cell Proliferation Promotion Effect of Hydrogel

L929 cells were seeded on the circular specimens of different hydrogels at the density of 10^4^ cells per well, and were stimulated by 0, 100, 200 and 400 mV voltages for 10 min daily for 1 d, 3 d or 5 d. The culture medium was replaced after each electrical stimulation. The cell viability was determined by the CCK‐8 assay.^[^ ^24^ ^]^ The detailed procedures were shown in SI S2.5.

### Live/Dead Cell Staining

To obtain the cell distribution and status, L929 cells were seeded on the hydrogels at the density of 5000 cells per well. On the day 3 of cell culture, the cells were incubated with 1 mM calcein AM for 1 h, followed by the incubation with 1 µg mL^−1^ propidium iodide (PI) at 37 °C for 5 min. The cells were then imaged using a super‐resolution microscope (N‐SIM E Nikon, Japan).

### Experiments on Skin Wound Repairing Effect of Hydrogel

All animal procedures and surgical processes were conducted in strict accordance with the guidelines of the Animal Facility and Usage Committee of the Plastic Surgery Hospital of the Chinese Academy of Medical Sciences (Approval No. 2023 animal (100)). To evaluate the wound healing promotion ability of PEG‐CMCS/AG/MXene hydrogel combined with NIR and ES, a full‐thickness skin defect model was established on New Zealand rabbit. The wounds on the backs of rabbits were randomly divided into 6 groups, Tegaderm^TM^, Control, PEG‐CMCS/AG/MXene, PEG‐CMCS/AG/MXene/ES, PEG‐CMCS/AG/MXene/NIR and PEG‐CMCS/AG/MXene/NIR/ES, with a total of 7 parallel experiments. The Control group underwent the modeling process without any treatment. Other groups were treated with the different dressing and subjected to the corresponding treatments for a total of 14 days. The detailed procedures can be found in Figure S2.6 (Supporting Information). The wound tissues were collected on day 7 and 14, fixed in 10% formaldehyde, and subjected to Hematoxylin and Eosin (H&E) staining and Masson trichromatic staining for histological analysis. Immunohistochemical staining of CD31 (neovascularization marker), αSMA (Myofibroblast), and TNF‐α (tumor necrosis factor‐α) was also conducted on the wound tissues collected on day 14.

### Statistical Analysis

All data were expressed as mean ± standard deviation (SD). One‐way analysis of variance (ANOVA) was used to determine whether the difference was statistically significant (*p < 0.05, **p < 0.01, ***p < 0.001).

## Conflict of Interest

The authors declare no conflict of interest.

## Author Contributions

X.M.M. conceived the idea, designed the experiments, and wrote the paper. L.Z.L. and H.L. assisted chemical synthesis of hydrogels and data analysis. Q.Q.Z. and H.W. assisted antimicrobial experiments. X.Y.L. and Z.F.W. collected and analyzed the data. Y.Q.F. acquired funding to support the research. Y.C. supervised the entire project, acquired funding to support the research, and reviewed and approved the final manuscript.

## Supporting information

Supporting Information

## Data Availability

The data that support the findings of this study are available from the corresponding author upon reasonable request.
