# Supplementary material for: Construction and Performance Study of a Dual‐Network Hydrogel Dressing Mimicking Skin Pore Drainage for Photothermal Exudate Removal and On‐Demand Dissolution
Source: Adv Sci (Weinh). 2024 Jul 28;11(36):2403362. doi: 10.1002/advs.202403362 (PMC11423237; doi:10.1002/advs.202403362)
Supplement: Supplementary file 1 — Supporting Information [file ADVS-11-2403362-s001.docx]

***Supplementary Information***

**Construction and Performance Study of a Dual-Network Hydrogel Dressing Mimicking Skin Pore Drainage for Photothermal Exudate Removal, Healing Promotion, and On-demand Dissolution**

*Xiaoxiao Ma ^a^,* *Lizhi Lin ^a^, Hang Luo ^a^,* *Qianqian Zheng ^b^, Hui Wang ^c^, Xiaoyan Li ^a^, Zhenfei Wang ^a^,* *Yongqiang Feng ^d,^ * , Yu Chen ^a, c,^ **

*^a^ School of Medical Technology, Beijing Institute of Technology, Beijing 100081, China*

*^b^ Department of Polymer Science and Engineering, Zhejiang University, Zhejiang 310027, China*

*^c^ School of Materials Science and Engineering, Beijing Institute of Technology, Beijing 100081, China*

*^d^ Plastic Surgery Hospital of Peking Union Medical College and Chinese Academy of Medical Sciences, Beijing, 100144, China*

**Corresponding author.*

*Email address: sdfrank@126.com (Y. Feng); cylsy@163.com (Y. Chen)*

**S1. Experimental Section**

1 g of LiF was added to 20 mL of 9 M HCl solution, and stirred magnetically at room temperature for 5 min to obtain the fluorine-containing etching solution. Then 1 g of Ti_3_AlC_2_ was slowly added, and stirred at 35 °C for 24 h to obtain a strongly acidic black suspension.^[1]^ After the reaction, the mixture was centrifuged at 3500 rpm for 5 min, and washed until the pH exceeded 6. After being dried at 50 ℃ for 3 h, multilayer Ti_3_C_2_T_x_ powder was obtained and stored in oxygen-free and light-free condition. Then 25 mg of multilayer Ti_3_C_2_T_x_ powder was added to 20 mL deionized water. After ultrasonication for 1 h in an ice bath, the mixture was centrifuged at 8000 rpm/min for 1 h. The supernatant was collected to obtain a suspension of single or few-layer MXene (Ti_3_C_2_T_x_) with a concentration of 0.25 mg mL^-1^.

**S2. Test section**

**S2.1. X-ray diffraction (XRD) spectra**

XRD spectra were collected for Ti_3_AlC_2_ and Ti_3_C_2_T_x_ using a PERT Pro MPD diffractometer (PANalytical, Netherlands) with Cu Kα radiation (λ = 1.5418 Å) in the range of 10-80 °.

**S2.2****. Transmission electron microscopy characterization (TEM）**

After the ultrasonic of MXene suspension, it was diluted 10 times. And then appropriate amount of it was deposited on the copper network, and dried for 24 h to volatilize the water. The morphology of MXene was characterized using a JEM 2100F microscope (JEOL, Japan) to observe the dispersion of MXene layers.

**S2.3. Physicochemical characterization of hydrogels**

**S2.3.1. Swelling properties**

Hydrogel discs with a diameter of 13 mm and a thickness of 2 mm were immersed in PBS solution (10 mM, pH 7.4). They were retrieved from the PBS solution at specific time intervals, and surface moisture was removed using filter paper. The average value was obtained after three consecutive measurements. The calculation for equilibrium swelling ratio (*ESR*) is as follows:^[2]^

$\text{ESR(\%) =}\text{ }\frac{M_{t}-M_{0}}{M_{0}}\times100\%$ (S1)

Where *M_t_* is the weight of the hydrogel disc after PBS immersion, and *M_0_* is the initial weight of the hydrogel disc.

**S2.3.2. Photothermal reversible drainage test**

Hydrogel discs of PEG-CMCS/AG/MXene with a diameter of 8 mm and a thickness of 2 mm were placed on a testing board. The temperature inside the hydrogel was recorded by infrared thermal imager after 15 min irradiation of NIR at 808 nm with an intensity of 1.5 W cm^-2^. And the equilibrium mass of the hydrogel in real-time was continuously recorded. Three parallel measurements were taken for each set of values. The evaporation rate (*V_E_*) of the composite hydrogel is calculated as：

$\text{V}\text{E}\text{ }\text{=}\text{ }\frac{W-W_{0}}{S\times h}$ (S2)

Where *W* is the weight of the hydrogel disc after NIR irradiation, *W_0_* is the initial weight of the hydrogel disc, *S* is the area of the light source, and *h* is the duration of light exposure.

**S2.3.3. Conductivity measurement**

The resistance of hydrogel samples was measured by AC impedance method. The hydrogel was added to the 2 cm × 2 cm × 2 cm polytetrafluoron mold with electrodes inserted at both ends. The system was connected to an electrochemical workstation via wires, and the electrical resistance of the composite hydrogel was measured at frequencies ranging from 10^3^-10^5^ Hz. The conductivity (*σ*) was calculated as follows:^[3]^

$\sigma= \frac{L}{RA}$ (S3)

Where *σ* is the conductivity, *L* is the thickness of the hydrogel, *A* is the contact area between the stainless steel electrode and the composite hydrogel, and *R* is the resistance measured after applying a current.

**S2.4. *In vitro* biological performances**

**S2.4.1. *In vitro* antibacterial activity**

Luria-Bertani (LB) liquid culture medium was composed of 10 g L^-1^ tryptone, 10 g L^-1^ sodium chloride, and 5 g L^-1^ yeast extract. The LB solid culture medium was prepared by adding 15 g agar powder to 1 L of LB liquid culture medium.^[4]^ The culture medium should be sterilized by autoclaving at 121 ℃ for 20 min in a high-pressure steam sterilizer before use. After sterilization, it was poured into sterile petri dishes with a diameter of 15 cm for using.

0.2 g samples were brought into contact with 20 μL of *E. coli* and *S. aureus*, respectively with a concentration of 10^6^. All samples were divided into groups with/without laser irradiation. In the irradiation group, the bacterial solution was exposed to the hydrogel sample under the NIR laser (808 nm, 1.5 W/cm^2^) for 10 min. The samples containing the bacterial suspension were incubated in a 37 ℃ constant temperature incubator for 2 h. Afterward, they were taken out and resuspended in 980 μL of PBS buffer. The resuspended solution was diluted to 10 times, 100 times, and 1000 times, respectively, and then 100 μL was spread on LB solid culture medium.^[4]^ The plates were incubated at 37 ℃ for 18 h to record the colony growth. The antibacterial rate is calculated using the following formula:

$Antibacterial rate (\%) = \frac{N_{x}-N_{0}}{N_{0}}\times100\%$ (S4)

Where *N_x_* is the number of bacterial colonies on the agar plate with PEG-CMCS/AG/MXene (75, 125, 175, 250), and *N_0_* is the number of bacterial colonies on the agar plate with PEG-CMCS/AG.

**S2.4.2. Hemolysis rate test**

Fresh rabbit blood was collected and centrifuged at 1800 rpm at 25 ℃ for 10 min to obtain red blood cells. The red blood cells were then washed with PBS until the supernatant became clear. The red blood cell solution was diluted to a 2 % standard dispersion (v/v). Subsequently, hydrogels were added to 2 % standard dispersion at different concentrations (20, 40, 60, 80, 100 mg/mL) and incubated in a water bath at 37 ℃ for 2 h. PBS and 30 μL of Triton X-100 (polyethylene glycol octylphenyl ether) were used as negative and positive controls, respectively. After 2h incubation, the solution was centrifugated at 1800 rpm for 10 min and the hemolysis was recorded with photographs. The supernatant was collected and added into the 96-well plate with 100 μL per well. The absorbance (A) values at wavelength of 540 nm were measured using a microplate reader. Hemolysis rate was calculated using the following formula:^[5]^

$\text{H}\text{emolysis rate}\text{ (}\text{\%}\text{) }\text{ }\text{=}\text{ }\frac{A_{s}-A_{n}}{A_{p}-A_{n}}\times100 \%$ (S5)

Where *A_s_* is the OD value of the sample, *A_n_* is the OD value of the negative control, and *A*_p_ is the OD value of the positive control.

**S2.4.3. Cell toxicity assessment**

The activity of L929 cells was measured by CCK-8 method.^[6]^ The gel was immersed in the complete medium at the concentration of 10 g mL^-1^ for 24 h, and the impurities in the extract were filtered by 0.22 μm filter head. After cell adhesion, the culture medium was removed, and the cells were cultured in the extraction solution at 37 °C for 1 d, 3 d, and 5 d, respectively. Cell viability was determined using the CCK-8 reagent. A coloration solution was prepared by mixing complete culture medium with CCK-8 reagent with 1:9 ratio. In a 96-well plate, 100 μL of the coloration solution was added to each well. After being incubated for 2 h, the absorbance at 450 nm (OD value) was measured using a microplate reader. The cell viability is calculated by the following formula:^[7]^

$Cell viability \left（ \% \right） = \frac{A_{\mathrm{ex}}-A_{0}}{A_{control}-A_{0}}\times100\%$ (S6)

Where *A_ex_* represents the OD value after incubation of L929 cells, hydrogel extraction solution, and CCK-8; *A_control_* represents the OD value after incubation of L929 cells, complete culture medium, and CCK-8; and *A_0_* represents the OD value after incubation of complete culture medium with CCK-8.

**S2.5. *In vitro* detection of hydrogel-mediated enhancement of cell proliferation**

The circular hydrogel samples with a diameter of 8 mm and a thickness of 2 mm were washed with PBS solution and subjected to 30 min of ultraviolet sterilization. They were then immersed in a 48-well plate containing complete medium and soaked at 37 ℃ for 24 h to achieve swelling equilibrium. L929 cells were then seeded onto the circular hydrogel specimens at a density of 5000 cells per well. After 24 h of incubation, it was confirmed that the cells had successfully grown onto the hydrogel samples under an optical microscope. Subsequently, they were subjected to constant voltage stimulation at 0, 100, 200, and 400 mV, respectively, for 1, 3, and 5 days with 10 min a day. The culture medium was replaced daily after each electrical stimulation.^[8]^ Finally, 0.5 mL medium containing 10% CCK-8 reagent was added to each well to measure cell viability. The OD values of the solution at 450 nm were measured by enzyme-labeled instrument. To ensure the validity of the experimental data, all samples were tested for 5 replicates and the averaged results were used.

**S2.6. Determination of skin wound repairing effect of hydrogel**

After anesthesia, 0.5 mm deep wounds with the diameters of 1 cm were created on the back of New Zealand rabbits using a punch. The wounds were randomly divided into 6 groups: Tegaderm^TM^, Control, PEG-CMCS/AG/MXene, PEG-CMCS/AG/MXene/ES, PEG-CMCS/AG/MXene/NIR, and PEG-CMCS/AG/MXene/NIR/ES, with a total of 7 parallel experiments. The Control group was left untreated after the wound created. The PEG-CMCS/AG/MXene/ES group was subjected to electrical stimulation for 10 min every day. The PEG-CMCS/AG/MXene/NIR group was treated under 808 nm laser irradiation for 10 min every day. The PEG-CMCS/AG/MXene/NIR/ES treatment involved 10 min electrical stimulation and 10 min irradiation with an 808 nm laser on the hydrogel every day. The wound was treated and observed every other day. Photographs were taken to record the wound area and Image J software was utilized to measure the wound area. The formula of wound healing (*WH*) is calculated as follows:

$\text{WH}\text{ (\%)}\text{ }\text{=}\text{ }\frac{A_{0}-A_{n}}{A_{0}}\times100\%$ (S7)

Where *A_0_* represents the initial wound area, *A_n_* represents the wound area on days 0, 4, 7, and 14 after modeling.

**S3. Results**

**Figure S1.** XRD spectra of Ti_3_AlC_2_ and Ti_3_C_2_T_x_

**
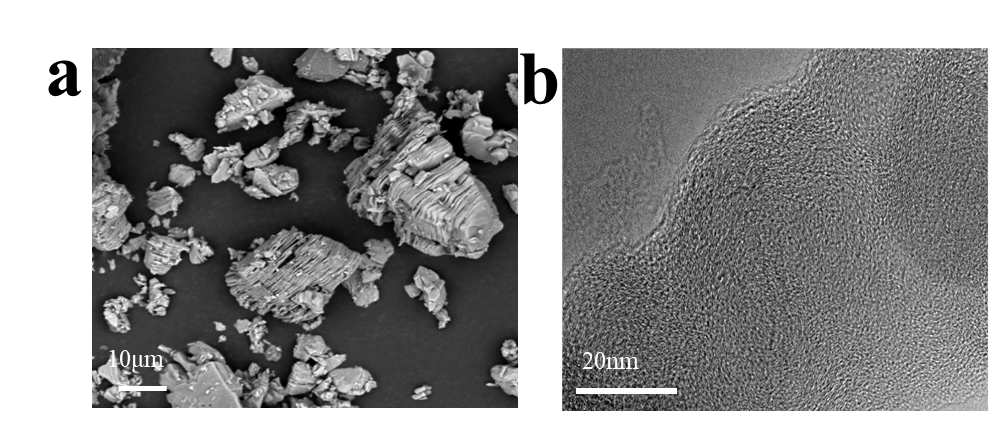
Figure S2.** Morphological characterization of MXene. (a) SEM image of multilayer Ti_3_C_2_T_x_; (b) TEM images of single layer or few layers Ti_3_C_2_T_x_.

**
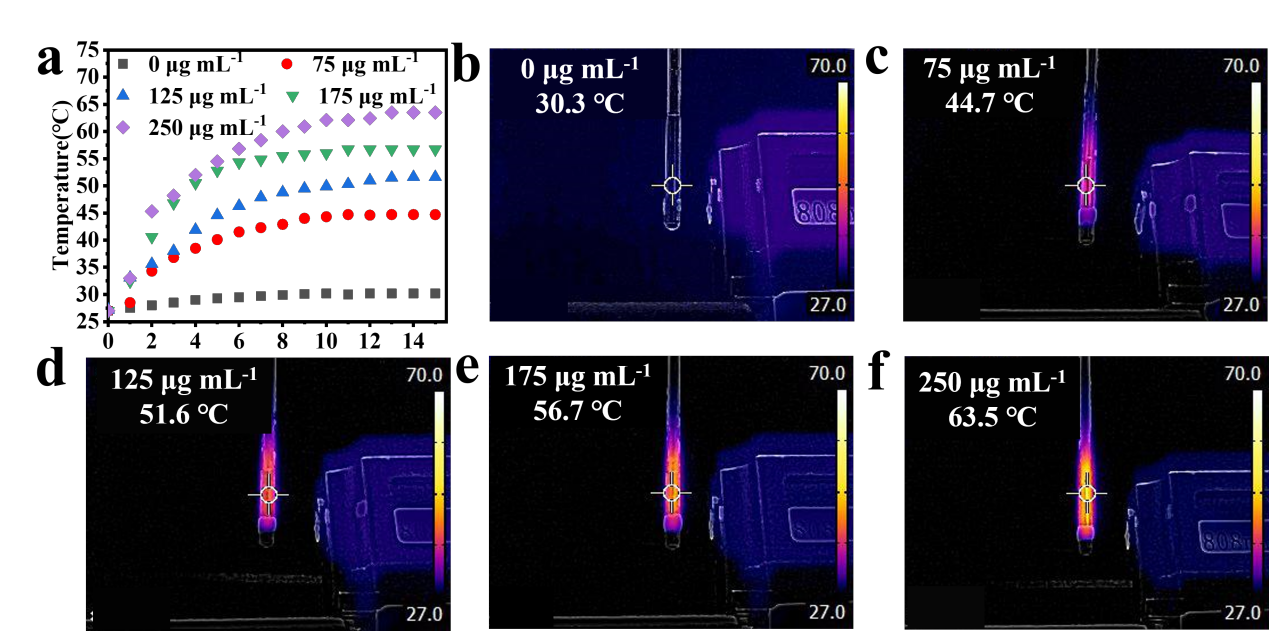
**

**Figure S3.** Physicochemical characterization of MXene. (a-f) Temperature changes of MXene during 15 min of irradiation with an 808 nm laser (1.5 W cm^−1^) (a) and the infrared thermographic images (b-f) after the irradiation.


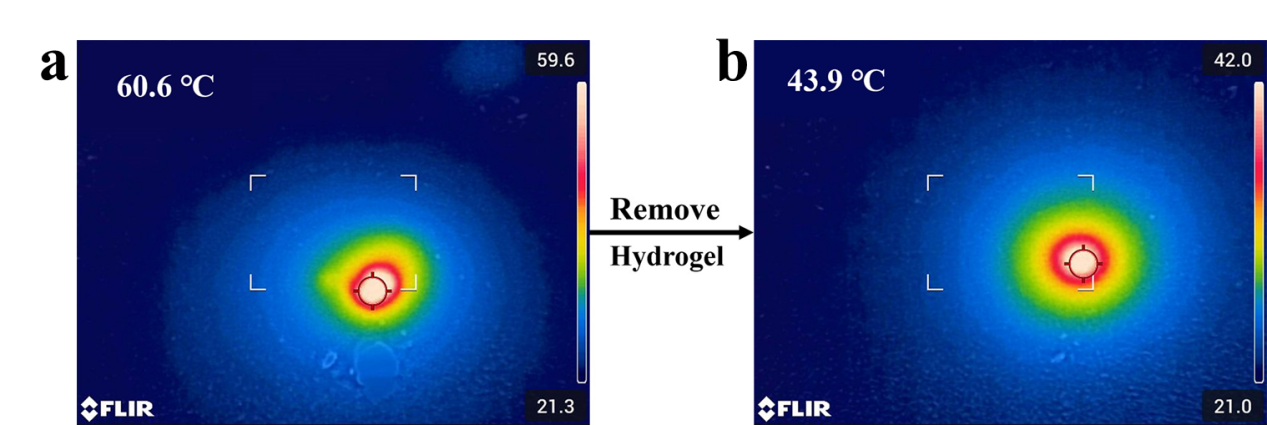


**Figure S4.** Comparison between the temperature inside the hydrogel (a) and the temperature actually reaching the skin (b) after near infrared light irradiation.


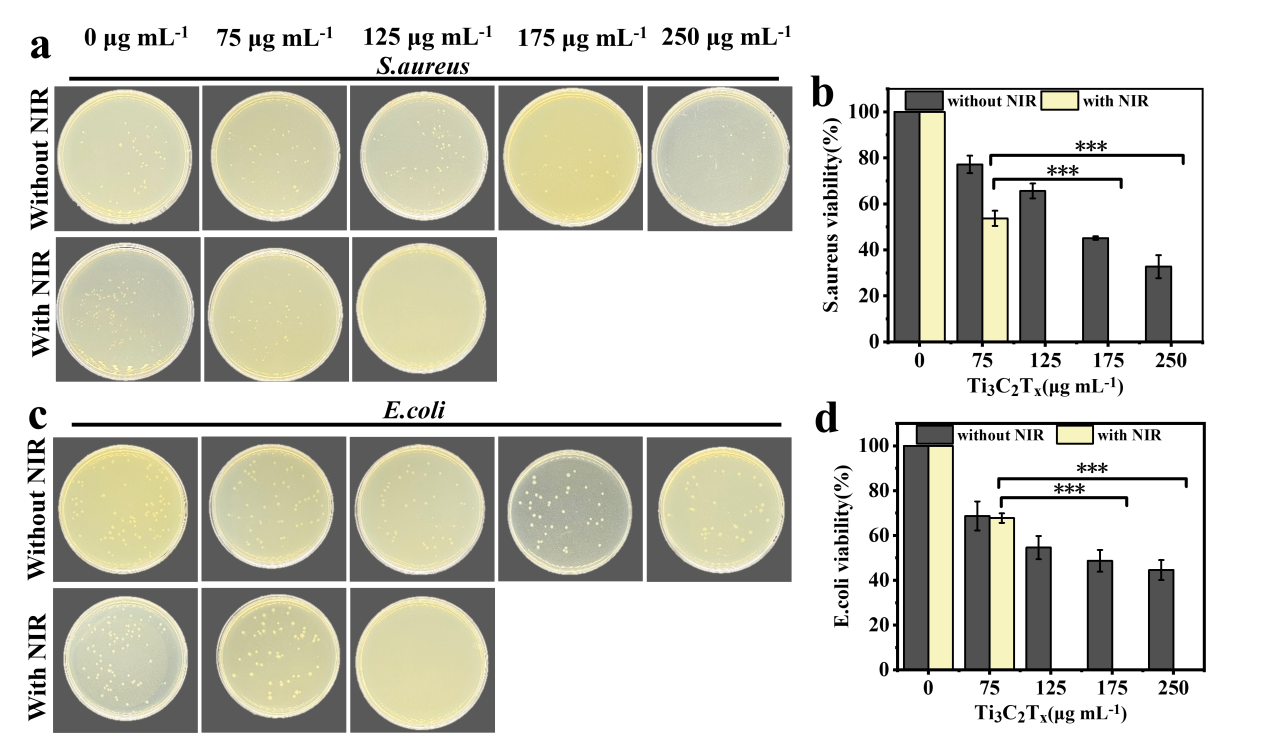


**Figure S5.** *In vitro* biological characterization of MXene. (a, b) Images and quantitative analyses of *S. aureus* on MXene with/without NIR irradiation; (c, d) Images and quantitative analyses of *E. coli* on the MXene with/without NIR irradiation.

**References**

[1] Z. Qi, S. Wang, Y. Li, L. Wang, L. Zhao, Q. Ge, J. Z. Zhang, *Ceram. Int.* **2021**, *47*, 16555.

[2] J. Tang, T. Katashima, X. Li, Y. Mitsukami, Y. Yokoyama, N. Sakumichi, U. Chung, M. Shibayama, T. Sakai, *Macromolecules* **2020**, *53*, 8244.

[3] C. Lee, H. Wu, Y. Hu, M. Young, H. Wang, D. Lynch, F. Xu, H. Cong, G. Cheng, *ACS Appl. Mater. Interfaces* **2018**, *10*, 5845.

[4] F. Wu, H. Zheng, W. Wang, Q. Wu, Q. Zhang, J. Guo, B. Pu, X. Shi, J. Li, X. Chen, W. Hong, *Science China Mater.* **2021**, *64*, 748.

[5] M. S. Bakshi, *Chem. Res. Toxicol.* **2017**, *30*, 1253.

[6] S. D. Liu, D. S. Li, Y. Wang, G. Q. Zhou, K. Ge, L. Jiang, D. N. Fang, *Biomater. Sci.* **2022**, *10*, 3585.

[7] J. Yang, Y. Chen, L. Zhao, Z. Feng, K. Peng, A. Wei, Y. Wang, Z. Tong, B. Cheng, *Compos. B Eng.* **2020**, *197*, 108139.

[8] L. Mao, S. Hu, Y. Gao, L. Wang, W. Zhao, L. Fu, H. Cheng, L. Xia, S. Xie, W. Ye, Z. Shi, G. Yang, *Adv. Healthc. Mater.* **2020**, *9*, 2000872.
